# The Veterinarian as a Naturalist1A paper presented before the Louisiana Society of Naturalists, at New Orleans, April 7, 1899.

**Published:** 1899-06

**Authors:** W. H. Dalrymple

**Affiliations:** Baton Rouge, La.


### THE VETERINARIAN AS A NATURALIST.[1]

By W. H. Dalrymple, M.R.C.V.S.,
BATON ROUGE, LA.

(Concluded from page 271.)

The second section of the zoöparasites requiring our attention—the entozoa, vermes, or helminths—is made up of an immense collection, and is usually divided into two classes, viz., the plathelminths, with bodies generally flat, and the nemathelminths, the bodies of which are nearly cylindrical.

The plathelminths comprise the following three orders: Cestodes, trematodes, and turbellaries. The first two, however, are limited to a parasitic existence. To the first of these belong the tæniæ or tapes, and their life-histories form an interesting study from the stand-point of a naturalist as well as that of the pathologist. In the adult stage they exist in the intestines of the higher animals, but in the immature stages they undergo a certain number of metamorphoses and migrations, which are often brought about in the

[1] A paper presented before the Louisiana Society of Naturalists, at New Orleans, April 7, 1899.

most diverse organs of different hosts. It may be of interest to note a few of the tapes of the various domestic animals, and it may be stated that although a number of these plathelminths are known in the adult stage to be peculiar to different animals, this is not the case in every instance with regard to their cystic or hydatid form.

In the alimentary canal of the equidæ three species of tænia have been observed, but, so far, nothing whatever is known of their cystic form. These are the tænia plicata, the tænia mamillana, and the tænia perfoliata.

Cattle also have three species, and like those of the equidæ they are entirely unknown in their cystic form. They are the tænia alba, the tænia expansa, and the tænia denticulata.

It is said that after the dog the sheep most frequently harbors the greatest number of tæniæ in its intestines. Those of this ruminant belong to eight distinct species, all of their cystic forms being unknown. They are as follows: The tænia expansa and the tænia alba, both of which are common to the ox and sheep; the tænia fimbriata, the tænia benedeni, the tænia vogti, tænia ovilla, tænia centripunctati, and tænia globipunctata.

It seems rather wonderful to remark that although the hog harbors a large number of intestinal parasites, up to the present, according to Neumann, no adult form of cestode has been observed in it.

When we come to the dog, however, it is said of him that he is the favorite host of tapeworms, eight species being put down as his share, and of which the greater number of the cystic forms are known. The tænia serrata has its cystic form in the cysticercus pisiformis, frequently found in the peritoneal cavity of hares and domestic or wild rabbits; the tænia marginata, whose cystic form is the cysticercus tenuicollis, found in the peritoneal cavity (and occasionally in the pleural cavity), more especially of domestic ruminants; the tænia echinococcus, cystic form of the echinococcus veterinorum, found in most of the organs of the herbivora and even in man, but more frequently observed in the liver and lungs of ruminants and the hog; the cystic form of the tænia cucumerina, or tænia canina, was long unknown, but discovered in 1869 in the body of the dog-louse, the trichodectes latus. The cystic form of the tænia litterata, I believe, is unknown. And the tænia coenurus, the hydatid form of which is developed in the cerebro-spinal cavity of the sheep.

The digestive canal of the cat affords asylum to three species of tænia: The tænia crassicollis, cystic form of the cysticercus fasciolaris, found in the liver of mice and of various kinds of rats: the tænia elliptica and the tænia litterata, the latter being confounded with the tænia of the dog bearing that name.

These may be said to be the principal tæniæ found in the domestic animals in the adult stage, but, as some hydatid forms found in the muscles of our meat animals develop into mature tapes in the human being, through the consumption of what is known as "measly flesh," it may be interesting as well as instructive from a public health stand-point to allude to two tæniæ which infest man. These are the tænia solium and the tænia mediocanellata or saginata. The cystic stage of the former of these parasites of man is the cysticercus cellulosæ, found in this form embedded in the muscular tissues of the hog. When this article of food is imperfectly cooked, or, at least, not sufficiently so to destroy the hydatid, the latter develops in the human digestive canal into the mature tape, the tænia solium. The second, the tænia mediocanellata or tænia saginata of the human family, has its cystic form, the cysticercus bovis, in the flesh of the ox tribe; and when this is partaken of in a somewhat rare condition, with the hydatids remaining possessed of vitality, tæniasis is produced, as in the former-mentioned case. This fact ought to give emphasis to the urgent necessity for careful meat-inspection by properly qualified individuals.

As the study of the entozoa is one of interest to the naturalist, I may be pardoned for pursuing the subject a little further.

The second variety of the plathelminths are the trematodes, and those of chief importance to the veterinarian belong to the suborder distomata. The principal species of distomes of the domestic animals infest the liver, and the hepatic form is, therefore, the most important, the first place being taken by the distoma hepaticum; the distoma lanceolatum, perhaps, taking a secondary position. The former species is pre-eminently a parasite of ruminants, and, as the name would indicate, is most frequently found in the bile-ducts. The lanceolatum is often observed along with the hepaticum in ruminating animals, but it has also been observed in the rabbit, hare, pig, ass, dog, cat, and man. We have also the distoma texinicum, found in Texas cattle, and described by the veterinarian of the Texas Experiment Station, Dr. Francis, a year or two ago. The life-history of these parasites is extremely interesting, but time will hardly permit of our taking it up at present.

Coming now to the second class, under the head of entozoa, we have the nemathelminths, or round worms, which comprise two orders. These are the acanthocephali and the nematodes. The former include only the echinorhyncus, which in the adult stage lives in the digestive canal of vertebrates; but the latter are numerous, and are found in all the organs of the domestic animals with the exception of the bones and the nervous system. The following are some of the principal nematodes of the various domesticated animals: In the horse we have the oxyuris curvula, found in the cæcum and colon; the ascaris megalocephala, probably the most common internal parasite of the horse; the spiroptera megastoma, found encysted in tumors in the walls of the stomach; the strongylus armatus, found in the bloodvessels, sometimes in the heart, but chiefly in the anterior mesenteric artery; the sclerostoma tetracanthum, found in the cæcum and colon; the eustrongylus gigas, found in the bladder, kidneys, and tissues of the perineal region; the filaria lachrymalis, found in the lachrymal duct, and the filaria papillosa, found in various locations in the body and occasionally occupying the anterior chamber of the eye.

Among the nematodes of the ox we find the following: The ascaris lumbricoides in the small intestine; the strongylus radiatus, ventricosus, and inflatus in the stomach and intestines; the strongylus micrurus in the trachea and bronchi; the eustrongylus gigas, filaria lachyrmalis, and papillosa, common also to the horse; and the spiroptera scutata œsophagea bovis, found in the œsophagus. This has been given a new genus by Stiles, of the Bureau of Animal Industry, viz., myzomimus, and the parasite the myzomimus scutata.

The sheep also harbors a number of round worms, the most common being the strongylus filaria, in the trachea, bronchial tubes, and parenchyma of the lung; the strongylus contortus, found in the abomasum; the strongylus filicollis and dochmius hypostomus in the intestines; and the tricocephalus affinis in the cæcum and sometimes in other portions of the intestines.

The pig or hog is the host of the following parasites of this class: The ascaris lumbricoides, sometimes named the ascaris suilla, found in the intestines; the tricocephalus crenatus, also in the intestines. The spiroptera strongylina in the walls of the stomach; the stephanurus dentatus in the adipose tissue around the kidney; the sclerostomum dentatum and the echinorhyncus gigas in the small intestines; the strongylus paradoxus in the lungs; and the trichina spiralis embedded in the muscles. This nematode, although one of the smallest of the intestinal parasites, requires more than merely a passing notice, as it is responsible for the disease in the human being known as trichinosis, trichiniasis, or "flesh-worm disease."

It exists in two distinct forms, viz., the partially developed or encysted, and the fully developed or intestinal. The following is a short history of the trichina: In a partially developed state it is found encysted in the muscles of the hog. Here it is sexually immature, but when the human subject partakes of the flesh imperfectly cooked the cyst wall is ruptured in the stomach, the embryo escapes, and in forty-eight hours it becomes sexually mature. Coition takes place, and in about eight days after entering the stomach young are born viviparously; they commence to migrate, entering the muscular tissues of the abdomen chiefly, and there become encysted. During migration they cause an amount of disturbance which is sometimes fatal in the human subject.

The following nematodes are found in the digestive canal of the dog: The ascaris marginata, spiroptora sanguinolenta, tricocephalus affinis, tricocephalus depressiusculus, and dochmius trigonocephalus. The tricosoma plica is found in the bladder; the filaria immitis in the heart and bloodvessels; and the filaria trispinulosa in the capsule of the crystaline lens.

I have not alluded to the intestinal parasites of poultry, but I might just mention that something like ten species of cestodes, seven trematodes, and about ten nematodes have been described.

The third section, or arthropodes, I will merely touch upon, as I have already occupied too much time. Of the four classes—crustacea, arachnidæ, myriapodes, and insects—the arachnidæ and insects alone contain species which are parasitic in the domestic animals. I cannot afford to go further into this section, as it would only lead me deeper into a subject with which, although of intense interest from a natural history stand-point, I have already taken up considerable time.

I must confess, Mr. President, that when I commenced this paper I felt extremely doubtful as to my ability to put enough into it to occupy more than ten or fifteen minutes' reading, but the subject is so comprehensive and expansive that as I became more interested in it there seemed to be much more difficulty in knowing just when to stop than what more to say. There is a great deal more that could be said, however, but I must now draw to a close. I must repeat what I said at the commencement: That the veterinarian, as a rule, has rarely an opportunity to specialize. The calls upon his services are of such a general and diverse character that he has to be "all things to all men," so to speak. He may be asked one minute to prescribe for a dyspeptic cat, the pride and joy of some fastidious old maid; the next to investigate, control, and eradicate an epizoötic of some contagious disease; and the next, perhaps, to perform some delicate piece of ocular or laryngeal surgery. To meet such varied demands he is compelled, however, to keep abreast of what is going on in the numerous branches of modern medicine and surgery. And, besides studying the nature of the animals with which he has to deal, he will, if he is wise, make a more or less careful study of human nature as well, for it is frequently the case that the owner is much more difficult to treat than is his animal.

I trust the Society will overlook any seeming tendency to veterinary professionalism on my part in the " make-up " of this paper. To convey such an impression is the farthest from my intention. What I desire is simply to show that with a curriculum of study made up of such subjects as those to which I have made brief allusion, and a great many more which I have not mentioned, if the modern graduate from our most reputable veterinary school is not somewhat of a naturalist, I think you will agree with me that he at least ought to be.

---

The spring meeting of the Pennsylvania State Board of Agriculture, held at Bloomsburg, was addressed on May 31st by State Veterinarian Leonard Pearson; subject, " The Work of the State Livestock Sanitary Board;" and by Dr. M. P. Ravenel, bacteriologist of the board, on the subject, " Bacteriology for the Farmer."

Dr. M. E. Conrad, of West Grove, Pa., represented Chester County on managers' day at the spring meeting of the Pennsylvania State Board of Agriculture on June 1, 1899.

Among the veterinarians at the Philadelphia open-air horseshow were to be noted Drs. F. H. Mackie and A. W. Clement, of Maryland; Drs. John W. Adams, A. F. Schrieber, S. J. J. Harger, George S. Fuller, Alexander Glass, and W. Horace Hoskins, of Philadelphia.

Dr. James C. Keely, formerly of Brownsville, Texas, has been ordered to report as an assistant inspector in the Bureau of Animal Industry to the Texas Fever Quarantine, Stockyard Station, Kansas City, Kansas.

Dr. A. E. Conrow, of Moorestown, N. J., recently lost by death his wife, and as the JOURNAL goes to press with the June number has himself been taken to the German Hospital, of Philadelphia, with the slight hope that surgical means may save his life from appendicitis.

